# G6PD deficiency as a underrecognized genetic risk factor for rare neurological disorders: evidence from a population-based genetic analysis

**DOI:** 10.3389/fgene.2026.1766081

**Published:** 2026-02-05

**Authors:** Qi Peng, Siping Li, Fen Lv, Xiaomei Zeng, Qingqiu Cheng, Baimao Zhong, Xiaomei Lu

**Affiliations:** 1 Laboratory Department, Dongguan Children’s Hospital, Dongguan, Guangdong, China; 2 Department of Medical and Molecular Genetics, Dongguan Institute of Pediatrics, Dongguan, Guangdong, China; 3 Key Laboratory for Children’s Genetics and Infectious Diseases of Dongguan, Dongguan, Guangdong, China

**Keywords:** Chinese population, G6PD deficiency, genetic risk factor, neurodevelopment, neurological disorders, pathogenic variants, whole-exome sequencing

## Abstract

**Background:**

Glucose-6-phosphate dehydrogenase (G6PD) deficiency is traditionally recognized as a risk factor for drug- or infection-induced hemolytic anemia. Emerging evidence implicates potential roles of G6PD in neurodevelopment, yet its association with rare neurological disorders remains underexplored in population-based genetic studies, especially within the Chinese population.

**Methods:**

We conducted a retrospective case-control study utilizing whole-exome sequencing (WES) data from a Chinese cohort. Six most prevalent pathogenic *G6PD* variants in China were screended in children with rare neurological disorders (n = 211) and in controls without neurological involvement (n = 202). Genotype and carrier frequency comparisons were performed. Stratified analyses were performed based on diagnostic certainty and the presence of *de novo* mutations. Multivariable logistic regression was employed to calculate sex-adjusted odds ratios (ORs) to control for potential sex-related confounding.

**Results:**

After adjusting for sex, the overall carrier rate of pathogenic G6PD variants was significantly higher in patients with neurological disorders than in controls (adjusted OR = 2.44, 95% CI: 1.18–5.06, p = 0.014). Further comparisons across specific groups revealed distinct patterns: affected male patients had a higher carrier rate than their own unaffected fathers (OR = 2.30, 95% CI: 1.08–4.91, p = 0.043), and mothers of case patients showed a higher carrier rate than mothers of controls (OR = 2.03, 95% CI: 1.09–3.78, p = 0.030). The variants NM_001042351.3: c.1376G>T (G6PD Canton) and NM_001042351.3:c.1388G>A (G6PD Kaiping) were the most prevalent across all groups.

**Conclusion:**

This population-based genetic analysis provides preliminary evidence that G6PD deficiency may be a underrecognized genetic risk factor for rare neurological disorders in Chinese children. The findings suggest a potential maternal genetic contribution and indicate that the phenotypic spectrum of G6PD deficiency may extend beyond hematological manifestations to include neurodevelopmental vulnerability. Important limitations include the lack of functional validation and the use of a clinical control group. Further prospective studies incorporating G6PD enzyme activity assessment and functional investigations are warranted to elucidate the underlying mechanisms.

## Introduction

Glucose-6-phosphate dehydrogenase (G6PD) deficiency is one of the most prevalent enzymopathies globally, affecting an estimated 400–500 million individuals worldwide (Nkhoma et al., 2009; Cappellini and Fiorelli, 2008; Yu et al., 2025). It is an X-linked disorder caused by pathogenic variants in the G6PD gene. These variants reduce enzyme activity and impair cellular defense against oxidative stress via the pentose phosphate pathway (PPP) (Meng et al., 2022). The classic clinical phenotype is characterized by acute hemolytic anemia triggered by oxidative insults such as certain drugs, infections, or specific foods (Bancone and Chu, 2021; Luzzatto et al., 2020; Boyadjian et al., 2022). Consequently, screening, diagnosis, and management have historically centered on its hematological manifestations.

Beyond its well-established role in erythrocyte homeostasis, emerging preclinical and clinical evidence suggests that G6PD may play a critical, yet underappreciated, role in neurological development and function (Tiwari, 2017; Merzon et al., 2023; Wang et al., 2025). The PPP is a primary cellular source of NADPH, essential for maintaining redox balance, synthesizing nucleic acids, and supporting antioxidant systems—processes crucial for the highly metabolically active and oxidatively vulnerable developing brain (Tang, 2019; TeSlaa et al., 2023; Fuentes-Lemus et al., 2023). Animal studies have demonstrated that G6PD deficiency can lead to embryonic neuronal apoptosis, impaired neural progenitor proliferation, and structural brain abnormalities (Merzon et al., 2023; Wang et al., 2025). Furthermore, epidemiological observations have hinted at potential associations between G6PD status and certain neurodevelopmental conditions, though population-based genetic evidence remains sparse and inconclusive (Merzon et al., 2023).

The diagnosis of G6PD deficiency traditionally relies on enzymatic activity assays. However, this method has significant limitations, including false negatives due to reticulocytosis, sample instability, and, critically, the challenge in accurately characterizing heterozygous females due to X-chromosome inactivation (Wang et al., 2014). Genetic testing, particularly for population-specific common pathogenic variants, offers a more reliable and direct assessment of carrier status, unaffected by hematological or physiological confounders. In the Chinese population, a spectrum of common pathogenic G6PD variants has been well characterized. Among these, six high-prevalence variants account for the majority of deficiency cases and are established as clinically significant: G6PD Canton (c.1376G>T, p.Arg459Leu), G6PD Kaiping (c.1388G>A, p.Arg463His), G6PD Gaohe (c.95A>G, p.His32Arg), G6PD QingYuan (c.392G>T, p.Arg131Leu), G6PD Viangchan (c.871G>A, p.Val291Met) and G6PD Chinese-5 (c.1024C>T, p.Leu342Phe) (RefSeq NM_001042351.3) (Peng et al., 2015; Liang et al., 2024; He et al., 2020; Li et al., 2023). Each of these variants is known to severely compromise enzymatic function and is consistently associated with markedly reduced G6PD activity in biochemical studies (Jiang et al., 2006; Tan et al., 2024).

Despite this background, a significant knowledge gap remains: Is G6PD deficiency, defined by the presence of these known pathogenic variants, a genetic risk factor for rare, early-onset neurological disorders in children? To address this question, we employed a retrospective genetic case-control strategy utilizing clinical whole-exome sequencing (WES) data. Our aims were to: (1) compare carrier frequencies of six common pathogenic G6PD variants between children with neurological disorders and non-neurological controls; (2) conduct within-family trio analyses to assess transmission patterns; (3) explore associations in relevant subgroups, including molecularly diagnosed cases and those with *de novo* mutations. This genetic epidemiology approach provides population-based evidence on the potential neurodevelopmental role of G6PD deficiency in Chinese children.

## Methods

### Study participants

We conducted a retrospective genetic case–control study. The study protocol was approved by the Ethics Committee of Dongguan Children’s Hospital. All participants were Han Chinese ethnicity.

The case cohort comprised 211 pediatric patients (aged <18 years) with heterogeneous rare neurological disorders who underwent diagnostic whole-exome sequencing (WES) at our hospital between January 2023 and November 2025. The primary diagnostic categories and their proportions were as follows: neurodevelopmental delay/intellectual disability (n = 114, 54.0%), epilepsy/epileptic encephalopathy (n = 25, 11.8%), autism spectrum disorder (n = 22, 10.4%), neuromuscular disorders (n = 20, 9.5%), and other rare neurological conditions (n = 30, 14.2%). The control group consisted of 202 age-matched pediatric individuals without neurological diseases who underwent clinical WES during the same period for other medical indications. The composition of the control group was as follows: urological/renal disorders (n = 75, 37.1%), hematological disorders (n = 32, 15.8%), immunological disorders (n = 38, 18.8%), and other conditions including congenital malformations, orthopedic diseases, and metabolic disorders (n = 57, 28.2%). All control individuals had no documented personal or family history of neurological/neurodevelopmental disorders.

### Genetic analysis

Genetic analysis was performed using whole-exome sequencing on the MGISEQ-2000 platform, achieving a mean coverage depth of >100× and >98% of the target region covered at ≥20×. Variant calling and annotation were conducted according to GATK best practices. In this study, we focused specifically on the six previously mentioned *G6PD* (NM_001042351.3) variants—c.1376G>T (p.Arg459Leu), c.1388G>A (p.Arg463His), c.95A>G (p.His32Arg), c.392G>T (p.Gly131Val), c.871G>A (p.Val291Met) and c.1024C>T (p.Leu342Phe), —which are known to be responsible for the majority of G6PD deficiency cases in the Chinese population. This targeted approach enabled a robust assessment of the disease burden attributable to these well-characterized mutations. Carrier status was defined as the presence of at least one variant allele, with hemizygosity assumed in male participants. Parents of probands were included where available for trio analysis to assess transmission patterns.

### Statistical analysis

Carrier frequencies were compared between cases and controls using chi-square or Fisher’s exact test, as appropriate. Odds ratios (OR) with 95% confidence intervals (CI) were calculated. To account for the significant sex imbalance between groups, multivariable logistic regression was performed to obtain sex-adjusted ORs. Subgroup analyses were performed by diagnostic certainty (confirmed vs. unknown etiology), and presence of *de novo* mutations in the proband. A two-sided P value of <0.05 was considered statistically significant. All analyses were performed using SPSS version 26.0.

## Results

### Participant characteristics

A total of 211 participants with neurological diseases (mean age: 5.56 ± 3.68 years; 74.4% male) and 202 non-neurological control subjects (mean age: 6.04 ± 4.60 years; 57.4% male) were enrolled in this study. No statistically significant difference was observed in age between the two groups (P > 0.05). Notably, the gender distribution differed significantly between groups (P = 0.001), with a higher proportion of males in the neurological-disease group. This demographic characteristic is relevant given the X-linked inheritance pattern of G6PD deficiency and the male preponderance often reported in neurodevelopmental disorder cohorts.

Among the neurological-disease cohort, 108 individuals (51.2%) obtained a definite molecular diagnosis, while 103 cases (48.8%) remained undiagnosed despite comprehensive evaluations. Additionally, *de novo* mutations were identified in 64 participants (30.3%) in the neurological-disease group. All aforementioned data, including statistical differences, are summarized in Table 1.

**TABLE 1:** Demographic and clinical characteristics of the neurological disease group and the non-neurological control group.

Characteristic	Neurological-disease group (N = 211)	Non-neurological control group (N = 202)	p value
Age (years), mean ± SD	5.56 ± 3.68	6.04 ± 4.60	P > 0.05
Sex, n (%)	​	​	0.001*
Male	157 (74.4)	116 (57.4)	​
Female	54 (25.6)	86 (42.6)	​
Diagnostic status, n (%)			
Definite molecular diagnosis	108 (51.2)	71 (35.1)	​
Undefined diagnosis	103 (48.8)	131 (64.9)	​
*De novo* mutation carrier	64 (30.3)	26 (12.9)	​

*The observed gender imbalance is consistent with the known epidemiology of the included neurodevelopmental conditions.

### Carrier frequency of pathogenic G6PD variants

We first investigated whether G6PD deficiency is enriched in children with rare neurological disorders. The overall carrier frequency of pathogenic G6PD variants was significantly higher among patients with neurological diseases than in controls (12.32% vs. 5.45%; odds ratio [OR] = 2.44, 95% confidence interval [CI]: 1.18–5.06, *p* = 0.014; Table 2A). In sex-stratified analyses, carrier frequency appeared higher in male patients compared with male controls (12.10% vs. 6.03%; OR = 2.15, 95% CI: 0.87–5.35; *p* = 0.104), though this difference was not statistically significant. Similarly, female patients showed a higher carrier rate than female controls (12.96% vs. 4.65%; OR = 3.03, 95% CI: 0.86–10.72; *p* = 0.106), which also did not reach statistical significance. Notably, the carrier rate among mothers of patients was significantly elevated compared with mothers of controls (15.64% vs. 8.42%; OR = 2.03, 95% CI: 1.09–3.78; *p* = 0.030). In contrast, no significant difference was observed between fathers of patients and fathers of controls (5.69% vs. 3.47%; OR = 1.68, 95% CI: 0.66–4.30; *p* = 0.350).

**TABLE 2:** Comparison of G6PD pathogenic variant carrier rates.

A. Between-group comparisons			
Comparison	Group1 (Neurological)	Group 2 (non-neurological)	Statistical analysis
All patients (adjusted)	12.32% (26/211)	5.45% (11/202)	**OR = 2.44** (95% CI: 1.18–5.06), χ^2^ = 6.05, p = 0.014*
Male patients	12.10% (19/157)	6.03% (7/116)	OR = 2.15 (95% CI: 0.87–5.35), χ^2^ = 2.64, p = 0.104
Female patients	12.96% (7/54)	4.65% (4/86)	OR = 3.03 (95% CI: 0.86–10.72), χ^2^ = 2.61, p = 0.106
Fathers	5.69% (12/211)	3.47% (7/202)	OR = 1.68 (95% CI: 0.66–4.30), χ^2^ = 0.88, p = 0.350
Mothers	15.64% (33/211)	8.42% (17/202)	**OR = 2.03** (95% CI: 1.09–3.78), χ^2^ = 4.74, p = 0.030*

B. Within-family comparisons			
Comparison	Proband group	Parent group	Statistical analysis
Neurological group			
Male probands vs. fathers	12.10% (19/157)	5.69% (12/211)	**OR = 2.30** (95% CI: 1.08–4.91), χ^2^ = 4.09, p = 0.043*
Female probands vs. mothers	12.96% (7/54)	15.64% (33/211)	OR = 0.80 (95% CI: 0.33–1.93), χ^2^ = 0.24, p = 0.624
Non-neurological group			
Male probands vs. fathers	6.03% (7/116)	3.47% (7/202)	OR = 1.79 (95% CI: 0.61–5.23), χ^2^ = 0.90, p = 0.343
Male probands vs. fathers	4.65% (4/86)	8.42% (17/202)	OR = 0.53 (95% CI: 0.17–1.67), χ^2^ = 1.01, p = 0.315

C. Subgroup analysis (neurological patients only)			
Comparison	Subgroup 1	Subgroup 2	Statistical analysis
Definitive vs. undefined diagnosis	15.74% (17/108)	8.74% (9/103)	OR = 1.96 (95% CI: 0.83–4.66), χ^2^ = 2.15, p = 0.142
*De Novo* mutation carriers vs. non-carriers	17.19% (11/64)	10.20% (15/147)	OR = 1.83 (95% CI: 0.81–4.16), χ^2^ = 1.74, p = 0.188

The analysis for “All Patients” was adjusted for sex. OR, odds ratio; CI, confidence interval. Bold values indicate statistical significance (p < 0.05).

### Case-parent comparisons

To explore potential parent-of-origin effects, we performed within-family analyses. In the neurological group, the carrier frequency was significantly higher among affected male probands than among their fathers (12.10% vs. 5.69%; odds ratio [OR] = 2.30, 95% confidence interval [CI]: 1.08–4.91; *p* = 0.043). In contrast, no significant difference was observed between affected female probands and their mothers (12.96% vs. 15.64%; OR = 0.80, 95% CI: 0.33–1.93; *p* = 0.624). Similarly, within the non-neurological (control) group, no significant differences were found between male probands and their fathers (6.03% vs. 3.47%; OR = 1.79, 95% CI: 0.61–5.23; *p* = 0.343) or between female probands and their mothers (4.65% vs. 8.42%; OR = 0.53, 95% CI: 0.17–1.67; *p* = 0.315). All within-family comparison results are summarized in Table 2B.

### Subgroup analysis of G6PD carrier frequency in neurological patients

Within the cohort of neurological patients, we performed subgroup analyses to investigate potential associations. No statistically significant differences in G6PD variant carrier frequency were observed between patients with a definitive diagnosis and those with an undefined diagnosis (15.74% vs. 8.74%; odds ratio [OR] = 1.96, 95% confidence interval [CI]: 0.83–4.66; *p* = 0.142; Table 2C). Similarly, the carrier rate among patients with *de novo* mutations was not significantly different from that among patients without such mutations (17.19% vs. 10.20%; OR = 1.83, 95% CI: 0.81–4.16; *p* = 0.188; Table 2C).

### Mutation spectrum of targeted G6PD variants

**FIGURE 1:**
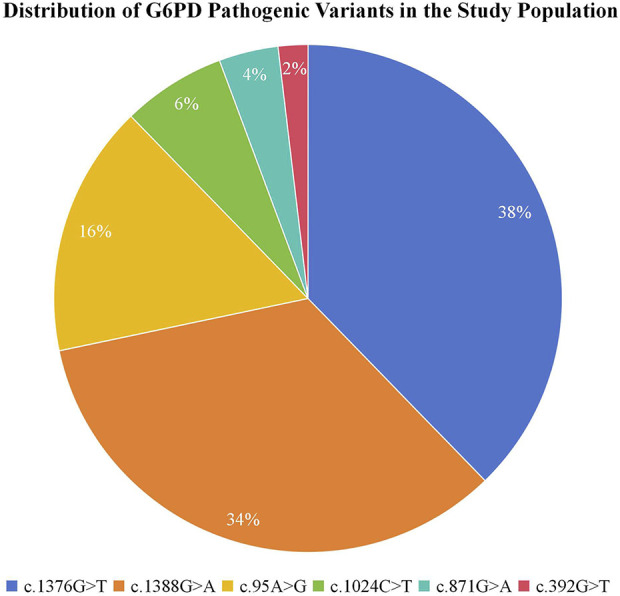
Distribution of six targeted pathogenic G6PD variants in the study cohort. The pie chart illustrates the relative frequencies of the six screened pathogenic G6PD variants among all mutant alleles detected across all participants (probands, controls, and parents). The most frequent variants were c.1376G>T (p.Arg459Leu; 38%) and c.1388G>A (p.Arg463His; 34%), which together accounted for 72% of all mutant alleles. The remaining variants—c.95A>G (p.His32Arg; 16%), c.1024C>T (p.Leu342Phe; 6%), c.871G>A (p.Val291Met; 4%), and c.392G>T (p.Gly131Val; 2%)—were less common.

We next characterized the spectrum of specific pathogenic variants contributing to the observed carrier burden. Among all study participants (probands, controls, and parents), analysis of the six targeted G6PD variants revealed that the c.1376G>T (p.Arg459Leu) variant was the most prevalent, accounting for 38% (40/106) of all detected pathogenic alleles. This was followed by c.1388G>A (p.Arg463His) at 34% (36/106) and c.95A>G (p.His32Arg) at 16% (17/106). The remaining variants—c.1024C>T (p.Arg342Cys), c.871G>A (p.Val291Met), and c.392G>T (p.Gly131Val)—were observed at lower frequencies of 6% (7/106), 4% (4/106), and 2% (2/106), respectively. The complete mutational spectrum is presented in Figure 1.

## Discussion

This retrospective analysis of WES data from a Chinese pediatric cohort provides an initial exploration of a potential association between pathogenic G6PD variants and neurological disorders. We observed a significantly higher carrier frequency of these specific G6PD variants among children with rare neurological diseases, as well as among their mothers, compared to control groups. These findings offer preliminary population-genetic evidence suggesting a potential role for G6PD deficiency in neurodevelopment.

The interpretation of these results is subject to several important limitations. First, the control cohort consisted of children who underwent WES for non-neurological indications and thus does not represent a healthy general population. This design may introduce unmeasured confounding biases. However, the absence of a significant difference in carrier rates between the fathers of the two groups—and the alignment of these rates with established local epidemiological data for G6PD deficiency (Peng et al., 2015; He et al., 2020)—serves as an internal validation. This partially mitigates concerns regarding major population stratification and suggests that the observed association is not merely an artifact of genetic background. Second, this study lacks functional validation. Our analysis was restricted to the genotypic detection of six known pathogenic variants, without corresponding measurements of G6PD enzymatic activity or biomarkers of oxidative stress in carriers, particularly in heterozygous females. For an X-linked gene like G6PD, where phenotypic expression in females is modulated by X-chromosome inactivation mosaicism, genotype alone is an insufficient proxy for functional phenotype.

Bearing our study’s limitations in mind, the proposed link between G6PD deficiency and neurodevelopmental disorders is mechanistically supported. Evidence from preclinical models consistently indicates that G6PD deficiency, by disrupting neural redox homeostasis, directly impairs neuronal and synaptic integrity.

For instance, Zhang et al. demonstrated that brain-specific G6PD deficiency in mice induced schizophrenia-like behaviors, including hyperactivity and cognitive deficits, concomitant with synaptic structural abnormalities, such as reduced dendritic spine density (Wang et al., 2025). Similarly, Shailja Singh elucidated that G6PD deficiency disrupts microglial function, leading to a pro-inflammatory state that creates a hostile microenvironment for neuronal survival (Mondal et al., 2025). Translating these mechanisms to human disorders, emerging clinical observations suggest an association to a certain degree. Eugene Merzon and colleagues conducted an epidemiological study proposing that the effects of G6PD deficiency are associated with ADHD development (Merzon et al., 2023). Furthermore, case reports, such as documenting G6PD deficiency in autistic boys from Saudi Arabia, alongside broader hypotheses that an altered redox microenvironment contributes to disorders like ADHD and ASD, offer preliminary correlative support (Al-Salehi and Ghaziuddin, 2009). Therefore, while our study provides early human cohort data, it aligns with and extends a convergent line of evidence from bench to bedside, underscoring that G6PD deficiency may be an underrecognized contributor to the complex etiology of neurodevelopmental disorders.

Given the central role of G6PD in maintaining cellular reductive capacity, and the observation in this study that maternal G6PD variant carriage was significantly elevated in the case group, a plausible hypothesis emerges. Specifically, altered systemic and/or reproductive tract redox status in carrier mothers may adversely shape the neurodevelopmental trajectory of their offspring, irrespective of the child’s own genotype, likely through impacts on the intrauterine or placental environment.

This proposed maternal redox-mediated effect receives partial support from prior experimental evidence. Studies in model organisms have demonstrated that G6PD deficiency or inhibition can compromise embryonic development (Nicol et al., 2000; Longo et al., 2002; Chen et al., 2017). This perspective further aligns with the Developmental Origins of Health and Disease (DOHaD) framework, which underscores the lasting influence of the maternal environment on offspring health (Hagemann et al., 2021). It is important to emphasize, however, that while our case-control data provide associative support, the proposed mechanisms remain testable hypotheses. Further investigation through animal models, placental studies, and well-designed prospective cohort research is warranted to establish causality and elucidate specific pathways.

Despite these limitations, the implications of this study are clear. It represents the first systematic description of G6PD variant carriage distribution in a sizable Chinese pediatric neurology cohort and suggests a maternally mediated effect worthy of deeper exploration. This finding argues for the incorporation of parental—particularly maternal—G6PD status into a broader diagnostic and counseling framework for families with a history of neurodevelopmental disorders. To address the shortcomings of the present work, future research should: 1) employ a case-control design with healthy, population-based controls; 2) integrate genetic data with functional phenotypes (e.g., enzyme activity, oxidative stress markers) and detailed clinical phenotypes (e.g., developmental scale scores, neuroimaging features), which is crucial for establishing causality; and 3) directly test the “maternal G6PD status affects offspring neurodevelopment” hypothesis in animal models to elucidate the underlying molecular and cellular mechanisms.

## Conclusion

In summary, this retrospective genetic association analysis suggests that carrier status for pathogenic G6PD variants—both in the child and the mother—is associated with an elevated risk of rare neurological disorders in Chinese children. However, the inherent limitations of this study, including potential control group selection bias and the absence of functional validation, preclude definitive conclusions regarding the underlying biological mechanisms. The primary contribution of this work lies in establishing a clear starting point and generating testable hypotheses for subsequent, more rigorous investigations. Ultimately, elucidating the precise role of G6PD in neurodevelopment will necessitate a multidisciplinary approach, integrating multi-omics data, functional experimentation, and detailed clinical phenotyping to move beyond observational genetic associations.

## Data Availability

The original contributions presented in the study are included in the article/supplementary material, further inquiries can be directed to the corresponding author.
